# Editorial: Enhanced Recovery After Surgery

**DOI:** 10.3389/fmed.2019.00062

**Published:** 2019-03-29

**Authors:** Ivana Budic, Ivan Velickovic

**Affiliations:** ^1^Department of Anesthesiology and Emergency Medicine, Medical Faculty, Clinical Centre Nis, University of Nis, Clinic for Anesthesiology and Intensive Care, Niš, Serbia; ^2^Department of Anesthesiology, State University of New York Downstate Medical Center (SUNY), Brooklyn, NY, United States

**Keywords:** enhanced recovery, protocols, surgery, anesthesia, intensive care

Enhanced Recovery After Surgery (ERAS) is a multidisciplinary approach to the care of surgical patient. ERAS protocols are multimodal perioperative care pathways designed to achieve early recovery after surgical procedures by maintaining preoperative organ function and reducing the profound stress response following surgery (1). ERAS started mainly with colorectal surgery but has been shown to improve outcomes in almost all major surgical specialties (2). ERAS process implementation involves a team consisting of surgeons, anesthesiologists, nutritionists, nurses, and other staff from services who are involved in patient care. In the past few years, several different centers have focused on ERAS programs resulting in many protocols that are now available with multiple elements to be considered (3). Despite the evidence of improved postoperative outcomes and recovery (4–7), ERAS implementation is slow and varies between different hospitals.

The overall goal of this research topic is to examine ERAS protocols and their implementation in different settings. The issue contains a series of review articles as well as original research articles.

Recent studies suggest that ERAS protocol can be successfully applied in vascular surgery. In a narrative review article, Stojanovic et al. present the evidence that application of ERAS program reduces the length of hospital stay, decreases the surgical, and non-surgical complications in the postoperative period, and improves the overall outcome in patients undergoing vascular surgery. Having reviewed recent literature, Dinic et al. highlight potential procedures and techniques that might be incorporated into the ERAS program after thoracic surgery. Golic et al. conducted a retrospective analysis of nine cases of patients who developed flail chest following blunt trauma, and were treated with early osteo-fixation of the chest wall and postoperative epidural analgesia. The authors concluded that surgical stabilization and epidural analgesia reduced ventilator support, shortened intensive care unit stay, and reduced medical costs. Vukovic and Dinic analyzed the components of ERAS protocols in urologic surgery. They concluded that there are still very few guidelines for ERAS protocols in urology and emphasize the importance of preoperative medical optimization, epidural analgesia, and nutritional management.

The idea of incorporating ERAS protocols in surgical intensive care unit (SICU) setting has great potential for promoting enhance recovery of SICU patients. Jovanović et al. comprehensively explain the role of sedation, analgesia, early oral intake, and early mobilization as integrative parts of SICU ERAS concept implementation.

Simić et al. in their review focus on the importance of postoperative analgesia in children. The authors describe the utility of continuous peripheral blocks (CPNB) for complete and prolonged postoperative analgesia of pediatric patients.

Sivevski et al. provide an in-depth review of the available data from the literature as well as evidence-based recommendations considering the concept of low dose spinal anesthesia for patients undergoing gynecological surgery. An interesting result of the study is that there was no respiratory depression in geriatric patients receiving 100 μg of spinal morphine. This study also highlights the hemodynamic stability of elderly patients who received low-doses of intrathecal bupivacaine in combination with opioids.

Pujic et al. developed a survey tool of 22 questions with multiple choice answers that was sent by email to all hospitals in Serbia (4 university teaching hospitals and 45 general hospitals) that provide obstetric services. The questionnaire asked whether ERAS protocols had been formally adopted for surgical patients and about their use in patients undergoing cesarean delivery (CD). Responses were obtained from 46 of 49 hospitals (3 university and 43 general hospitals; a 94% response rate). ERAS protocols were in use in 11 of 46 (24%) of surveyed hospitals and 63% of the time the responsibility for patients counseling was shared between the obstetrician and anesthesiologist. However, even ERAS hospitals reported a higher number of discharges after 3 days compared to surveys of UK hospitals where the majority of women are discharged within 2 days of their CD.

Quadratus lumborum block (QLB) is a new form of the abdominal wall block which is relatively easily performed thanks to clear ultrasound anatomic markers. QLB is safe and has found its place in multimodal postoperative pain therapy in patients undergoing abdominal surgery, gynecological and obstetric procedures, and orthopedic interventions on hips, whether interventions are performed in general or spinal anesthesia, both in adults and in children. Akerman et al. conclude that improved early oral intake and early mobilization can be more easily achieved with good pain control hereof QLB has a great potential in this area of ERAS.

In summary, this issue discusses particular aspects of ERAS protocols and an anesthesiologist's role in preoperative, intraoperative, and postoperative strategies for different surgical interventions and in different patient groups.

## Author Contributions

IB and IV have made a substantial contribution to the work and approved the final version of the manuscript to be published.

### Conflict of Interest Statement

The authors declare that the research was conducted in the absence of any commercial or financial relationships that could be construed as a potential conflict of interest.

## References

[1] Melnyk M Casey RG Black P Koupparis AJ. Enhanced recovery after surgery (ERAS) protocols: time to change practice? Can Urol Assoc J. (2011) 5:342–8. 10.5489/cuaj.693 22031616 PMC3202008

[2] Ljungqvist O Scott M Fearon KC. Enhanced recovery after surgery: a review. JAMA Surg. (2017) 152:292–8. 10.1001/jamasurg.2016.4952 28097305

[3] Taurchini M Del Naja C Tancredi A. Enhanced Recovery After Surgery: a patient centered process. J Vis Surg. (2018) 4:40. 10.21037/jovs.2018.01.20 29552522 PMC5847857

[4] Grant MC Gibbons MM Ko CY Wick EC Cannesson M Scott MJ. Evidence review conducted for the AHRQ Safety Program for Improving Surgical Care and Recovery: focus on anesthesiology for gynecologic surgery. Reg Anesth Pain Med. (2019). [Epub ahead of print]. 10.1136/rapm-2018-100071 30737316

[5] Parikh RP Myckatyn TM. Paravertebral blocks and enhanced recovery after surgery protocols in breast reconstructive surgery: patient selection and perspectives. J Pain Res. (2018) 11:1567–81. 10.2147/JPR.S148544 30197532 PMC6112815

[6] Rove KO Edney JC Brockel MA. Enhanced recovery after surgery in children: promising, evidence-based multidisciplinary care. Paediatr Anaesth. (2018) 28:482–92. 10.1111/pan.13380 29752858

[7] Tejedor P Pastor C Gonzalez-Ayora S Ortega-Lopez M Guadalajara H Garcia-Olmo D. Short-term outcomes and benefits of ERAS program in elderly patients undergoing colorectal surgery: a case-matched study compared to conventional care. Int J Colorectal Dis. (2018) 33:1251–8. 10.1007/s00384-018-3057-z 29721734

